# Biomechanical stress profiling in coronary arteries via two-phase blood FSI

**DOI:** 10.1007/s10237-025-02012-y

**Published:** 2025-09-22

**Authors:** Farajollah Zare Jouneghani, Reza Ghomashchi, Marco Amabili, Mergen H. Ghayesh

**Affiliations:** 1 School of Electrical and Mechanical Engineering, University of Adelaide, Adelaide, South Australia 5005 Australia; 2 School of Engineering, Westlake University, Zhejiang Province, Hangzhou, China

**Keywords:** Biomechanics, Two-phase blood flow, Red blood cells, Coronary artery, Oscillatory shear index

## Abstract

This study focuses on the biomechanical stress determination of the left circumflex (LCx) coronary artery reconstructed based on in vivo angiography images via the development of a comprehensive biomechanical model incorporating a two-phase two-way coupled three-dimensional fluid–structure interaction (FSI). The blood flow is modelled as a two-phase pulsatile fluid, with 45% red blood cells and 55% plasma, and the artery wall is modelled as a soft viscohyperelastic material that is able to dynamically react to the blood-induced pressure. The flow characteristics, such as pressure, velocity, phase distribution, near-wall haemodynamic parameters, and flow-induced indices, are determined. The von Mises stress (VMS) and the deformation field of the arterial wall are also obtained. Comparing results based on the two-phase FSI model and those of a single-phase FSI show that taking into account the red blood cells alters the stresses, providing a better understanding of potential cardiovascular events. In all the cases investigated in this study, the wall shear stress (WSS) levels predicted by the two-phase FSI model are consistently lower than those obtained from the single-phase simulations. For example, at the location of maximum WSS during peak systole, the single-phase simulation employing the Quemada viscosity model predicts 143.43 Pa, whereas the single-phase simulation based on the power-law model predicts 39.85 Pa. In contrast, the two-phase model yields a substantially lower value of 24.79 Pa.

## Introduction

Cardiovascular diseases (CVDs) rank as the leading cause of death worldwide, accounting for approximately 31% of all mortalities, equating to around 17.9 million people annually (Organisation 2023). Heart attacks and strokes alone are responsible for about 16% of deaths caused by CVDs, presenting not just a significant medical challenge for healthcare systems, but also a pressing social and economic issue (Naser et al. 2024; Organisation 2023). Understanding the potential cause of such diseases, risk factors, and main mechanisms underlying CVD leads to early diagnosis and consequently to choosing the most effective treatment strategies. Although medical imaging technology is advancing, it still falls short of fully investigating the complex interactions and correlations between heart structure and blood flow in the cardiovascular system. This is where progress in biomechanical engineering comes into play, providing a deeper understanding of the complex blood flow patterns and haemodynamics within the cardiovascular system. This knowledge complements clinical imaging methods such as ultrasound, MRI, CT, elastography, and X-ray angiography (Athani et al. 2024).

Numerical biomechanical studies on cardiovascular systems can be categorized into three main areas: computational fluid dynamics (CFD) (Malek et al. 2025; Maurya and Kumar 2025; Sahni et al. 2023; Su et al. 2014; Udupa et al. 2025; Wang et al. 2023), structural analysis (Alastrué et al. 2007; Buffinton and Ebenstein 2014; Cilla et al. 2012; Ghasemi et al. 2020; Lv et al. 2024; Melnikova et al. 2017; Mohammadi and Mequanint 2014; Noble et al. 2022), and fluid–structure interactions (FSI) (Chiastra et al. 2014; Fara et al. 2024; Gholipour et al. 2018, 2020; Nikpour and Mohebbi 2024; Qiao et al. 2023; Rostam-Alilou et al. 2022; Zhu et al. 2025a; Zhu et al. 2025b). Among these, the FSI method is an advanced approach in biomechanics, particularly for the heart and arteries, as blood flow exerts pressure on the arterial wall, causing deformation, or conversely, when the artery motion, caused by heartbeats, affects the blood flow (Carpenter et al. 2023). Experimental data obtained by using mock circulatory loops or mechanical characterization of the vessel’s wall are particularly important to develop realistic models (Amabili et al. 2019a; Amabili et al. 2020; Amabili et al. 2019b; Franchini et al. 2022, 2021).

Blood is a complex multiphase mixture mainly composed of plasma, red blood cells (RBCs), white blood cells (WBCs), and platelets, each with various volume fractions, including 55% plasma, 45% RBCs, and < 1% WBCs and platelets (Li et al. 2023; Morab et al. 2024). Among these, RBCs are the dominant component of circulating blood, playing an important role in physiological haemodynamics and the development of arterial pathologies (Valizadeh et al. 2024). RBCs are the main contributors to the frictional forces caused by blood on artery walls, which play a key role in determining blood viscosity (Michel and Martin-Ventura 2020); moreover, RBCs significantly contribute to the development of atherosclerosis, particularly when they accumulate in the areas of the arteries with curves or bifurcations (Tziakas et al. 2010). The majority of previous studies (Asanuma et al. 2013; Moghadasi et al. 2024; Wang et al. 2024) mainly hypothesized blood as a *single-phase* fluid to investigate its haemodynamic factors, while in reality, blood is a multiphase fluid. These single-phase models basically lack estimating the volume concentration of blood’s main components that affect its rheological behaviour, as well as predicting particle accumulation, which is one of the main initial steps towards atherosclerosis. To overcome this limitation, considering blood as a multiphase fluid in haemodynamic studies helps achieve a deeper understanding and, ultimately, a more accurate method of diagnosing heart-related diseases. Studies that considered blood as a multiphase fluid are limited: for instance, Athani et al. (Athani et al. 2021) developed an FSI model in which blood was considered as a two-phase flow, while the arterial wall was assumed to be isotropic and linearly elastic. Their study focused on analysing the impact of varying degrees of stenosis in the left anterior descending, LAD, and left circumflex, LCx, artery branches on haemodynamic variables. Ling et al. (Ling et al. 2021) used the CFD method to investigate the blood flow in a simplified geometry of a bifurcation channel by using a blood multiphase model. Further investigations on the analysis of coronary arteries considering blood as a multiphase flow can be found by Elhanafy et al. (2024); Giannokostas et al. (2022); Kopernik and Tokarczyk (2019); Melka et al. (2018); Owen et al. (2021); and Rongchang et al. (2024).

To date, only a limited number of studies have attempted to provide a comprehensive 3D biomechanical model of coronary arteries to address both blood haemodynamics and arterial stress analysis. Most of these studies have analysed the stress distribution in coronary arteries based on several assumptions, primarily using an idealized arterial model, treating blood as a single-phase fluid, and sometimes neglecting the blood flow and arterial wall interactions. Taking into account all of these factors together is computationally expensive. This study, for the first time, aims to present a comprehensive biomechanical model of the LCx coronary artery reconstructed based on in vivo images using a *two-phase blood comprised of plasma and RBCs* within a *two-way FSI* approach, allowing for taking blood flow dynamics and arterial wall deformation into consideration simultaneously. As will be shown, this changes the stress profiles and, hence, the stress-based predictions of coronary artery failure.

## Methodology

### Blood characteristics via multiphase governing equations

The kinetic theory of granular flow model (Campbell 2006; Homsy 1995) is employed with the primary phase being plasma (55%) and the secondary phase treated as a solid granular phase; only RBCs with a volume fraction of 45% are considered as the secondary phase, as they predominantly control the blood rheology, while other components are neglected due to their volume fractions being less than 1% (Brown et al. 2001; Jung et al. 2006a, 2006b). The kinetic theory-based two-phase blood flow model, as developed by Gidaspow (Gidaspow 1994), is highly applicable as it offers a comprehensive understanding of phase interactions, RBCs distribution, and effectively predicts phenomena influenced by blood components, such as the Fåhræus-Lindqvist effect (Arastoopour et al. 2022). In the kinetic theory of granular flow model, the viscosity of the red blood cell phase is computed using the random kinetic energy of the red blood cells equation, called granular temperature (Arastoopour et al. 2022). The basic mass and momentum balances for each component of blood (i.e. plasma and RBCs) are summarized as (Ansys® Fluent, Release 2021, Help System, Multiphase Model, ANSYS, Inc.)

1 $$\frac{\partial \left({\varepsilon }_{i}{\rho }_{i}\right)}{\partial t}+\nabla . \left({\varepsilon }_{i}{\rho }_{i}{\overrightarrow{v}}_{i}\right)=0 i\in \left[p,\text{RBC}\right],$$

2 $$\frac{\partial ({\varepsilon }_{p}{\rho }_{p}{\overrightarrow{v}}_{p})}{\partial t}+\nabla . \left({\rho }_{p}{\varepsilon }_{p}{\overrightarrow{v}}_{p}{\overrightarrow{v}}_{p}\right)=-{\varepsilon }_{p}\nabla P+{\rho }_{p}{\varepsilon }_{p}\overrightarrow{g}+\nabla .{\overline{\overline{\tau }}}_{p}+\beta \left({\overrightarrow{v}}_{\text{RBC}}-{\overrightarrow{v}}_{p}\right),$$

3 $$\frac{\partial ({\varepsilon }_{\text{RBC}}{\rho }_{\text{RBC}}{\overrightarrow{v}}_{\text{RBC}})}{\partial t}+\nabla . \left({\rho }_{\text{RBC}}{\varepsilon }_{\text{RBC}}{\overrightarrow{v}}_{\text{RBC}}{\overrightarrow{v}}_{\text{RBC}}\right)=-{\varepsilon }_{\text{RBC}}\nabla P+{\rho }_{\text{RBC}}{\varepsilon }_{\text{RBC}}\overrightarrow{g}-\nabla .{P}_{\text{RBC}}+\nabla .{\overline{\overline{\tau }}}_{\text{RBC}}+\beta \left({\overrightarrow{v}}_{p}-{\overrightarrow{v}}_{\text{RBC}}\right),$$

where $\rho$ is the density, $\overrightarrow{v}$ denotes the velocity, $\mu$ is the viscosity, $\overrightarrow{F}$ is the body force, $P$ is the pressure, $\overline{\overline{\tau }}$ denotes the stress tensor, and $\beta$ is the interface momentum exchange coefficient. The granular viscosity of the solid phase is calculated by following the granular temperature equation. The conservation equation of the granular temperature for RBCs is expressed as (Huang et al. 2009)

4 $$\frac{3}{2}\left[\frac{\partial ({\rho }_{\text{RBC}}{\varepsilon }_{\text{RBC}}\theta )}{\partial t}+\nabla .{(\varepsilon }_{\text{RBC}}{\rho }_{\text{RBC}}\theta {\overrightarrow{v}}_{\text{RBC}})\right]=\left(-{P}_{\text{RBC}}\overline{\overline{I}}+{\overline{\overline{\tau }}}_{\text{RBC}}\right):\nabla {\overrightarrow{v}}_{\text{rbc}}+ \nabla .\left({k}_{s}\nabla \theta \right)-\gamma +\varnothing ,$$

where $\overline{\overline{I}}$ is a unit tensor, ${k}_{s}$ is the granular conductivity, $\gamma$ is the collisional energy dissipation, and $\varnothing$ denotes the energy exchange between solid and fluid phases. The solid stress tensor for plasma and RBC phases is expressed as

5 $${\overline{\overline{\tau }}}_{\text{RBC}}={\varepsilon }_{\text{RBC}}{\mu }_{\text{RBC}}\left(\nabla {\overrightarrow{v}}_{\text{RBC}}+\nabla {v}_{\text{RBC}}^{T}\right)+{\varepsilon }_{\text{RBC}}\left({\lambda }_{\text{RBC}}-\frac{2}{3}{\mu }_{\text{RBC}}\right)\nabla .{\overrightarrow{v}}_{\text{rbc}}\overline{\overline{I}},$$

6 $${\overline{\overline{\tau }}}_{p}={\varepsilon }_{p}{\mu }_{p}\left(\nabla {\overrightarrow{v}}_{p}+\nabla {\overrightarrow{v}}_{p}^{T}\right)-\frac{2}{3}{\varepsilon }_{p}{\mu }_{p}\nabla .{\overrightarrow{v}}_{p}\overline{\overline{I}},$$

where ${P}_{\text{RBC}}$ is the solid pressure, ${\mu }_{\text{RBC}}$ is the shear viscosity, and ${\lambda }_{\text{RBC}}$ is the bulk viscosity. Solid pressure can be written as (Gidaspow 1994; Huang et al. 2009)

7 $${p}_{\text{RBC}}={\varepsilon }_{\text{RBC}}{\rho }_{\text{RBC}}\theta +2{\rho }_{\text{RBC}}(1+e){\varepsilon }_{\text{RBC}}^{2}{g}_{0}\theta ,$$

where $\theta$ is the granular temperature, $e$ is the restitution coefficient, and ${g}_{0}$ is the radial distribution function that modifies the probability of collisions between particles in dense areas and is written as (Huang et al. 2009)

8 $$g_{0} = \left[ {1 - \left( {\frac{{\varepsilon_{{{\text{RBC}}}} }}{{\varepsilon_{{{\text{RBC}},\max }} }}} \right)^{{{\raise0.5ex\hbox{$\scriptstyle 1$} \kern-0.1em/\kern-0.15em \lower0.25ex\hbox{$\scriptstyle 3$}}}} } \right]^{ - 1} ,$$

where ${\varepsilon }_{\text{RBC},\text{max}}$ is the RBCs volume fraction at maximum packing. The bulk viscosity, which is expressed as a function of granular temperature, is defined as (Gidaspow 1994)

9 $$\lambda_{{{\text{RBC}}}} = \frac{4}{3}\varepsilon_{{{\text{RBC}}}} \rho_{{{\text{RBC}}}} d_{{{\text{RBC}}}} g_{0} \left( {1 + e} \right)\left( {\frac{\theta }{\pi }} \right)^{{{\raise0.5ex\hbox{$\scriptstyle 1$} \kern-0.1em/\kern-0.15em \lower0.25ex\hbox{$\scriptstyle 2$}}}} ,$$

in which ${d}_{\text{RBC}}$ is the RBC diameter. The shear viscosity is defined as (Gidaspow 1994)

10 $$\mu_{{{\text{RBC}}}} = \frac{4}{5}\varepsilon_{{{\text{RBC}}}}^{2} \rho_{{{\text{RBC}}}} d_{{{\text{RBC}}}} g_{0} \left( {1 + e} \right)\left( {\frac{\theta }{\pi }} \right)^{{{\raise0.5ex\hbox{$\scriptstyle 1$} \kern-0.1em/\kern-0.15em \lower0.25ex\hbox{$\scriptstyle 2$}}}} + \frac{{10\rho_{{{\text{RBC}}}} d_{{{\text{RBC}}}} \sqrt {\theta \pi } }}{{96\left( {1 + e} \right)g_{0} }}\left[ {1 + \frac{4}{5}g_{0} \varepsilon_{{{\text{RBC}}}} \left( {1 + e} \right)} \right]^{2} ,$$

where the first term of Eq. (10) is the kinetic part and the second term is the collisional part. The momentum exchange coefficient between the continuous and solid phases, RBCs, and plasma is determined by the Gidaspow drag model as (Gidaspow 1994)

11 $$\left\{\begin{array}{c}\beta =150\frac{{\varepsilon }_{\text{RBC}}(1-{\varepsilon }_{p}){\mu }_{p}}{{\varepsilon }_{p}{d}_{\text{RBC}}^{2}}+1.75\frac{{\rho }_{p}{\varepsilon }_{\text{RBC}}\left|{\overrightarrow{v}}_{\text{RBC}}-{\overrightarrow{v}}_{p}\right|}{{d}_{\text{RBC}}} {\varepsilon }_{p}\le 0.8 \\ \beta =\frac{3}{4}{C}_{D}\frac{{\varepsilon }_{\text{RBC}}{\varepsilon }_{p}{\rho }_{p}\left|{\overrightarrow{v}}_{\text{RBC}}-{\overrightarrow{v}}_{p}\right|}{{d}_{\text{RBC}}}{\varepsilon }_{p}^{-2.65} {\varepsilon }_{p}>0.8\end{array}\right.,$$

where the drag coefficient is given by

12 $${C}_{D}=\frac{24}{{\varepsilon}_{p}{\text{Re}}_{p}}\left[1+0.15{({\varepsilon }_{p}{\text{Re}}_{p})}^{0.687}\right],$$

in which ${Re}_{p}$ is the Reynolds number given by

13 $${Re}_{p}=\frac{{\rho }_{p}{d}_{\text{RBC}}\left|{\overrightarrow{v}}_{\text{RBC}}-{\overrightarrow{v}}_{p}\right|}{{\mu }_{p}}.$$

The simulation conditions and the properties of plasma and RBCs, which are assumed to be rigid spherical particles, are given in Table 1. In addition, patch initialization is done for the entire volume with RBCs volume fraction of 45% and a granular temperature of 0.0001 *m*^*2*^*s*^*−2*^ (Huang et al. 2009).

**Table 1:** Simulation conditions and material properties (Huang et al. 2009)

Parameter	Value
Plasma Density (*kg.m*^*−3*^)	1003
Plasma Viscosity (*kg.m*^*−1*^*. s*^*−1*^)	0.001
RBCs Diameter (*μm*)	8
RBC Density (*kg.m*^*−3*^)	1096
Restitution coefficient	0.99999
RBC packing limit	0.7
RBC Volume Fraction	45%
Wall restitution coefficient	0.9999
Specularity coefficient	0.6

### Arterial wall model using viscohyperelasticity

In this study, the artery wall is considered two-layered, first layer, consisting of intima plus transition zone between intima and media, while the second layer includes the media and adventitia (Amabili et al. 2021). Both layers are assumed to exhibit isotropic and viscohyperelastic properties. The hyperelasticity of the artery is modelled based on a strain energy density function (${W}_{\text{s}}$) as a function of the invariants of the right Cauchy-Green deformation tensor as expressed in Eq. (14). The strain energy density function of the Mooney–Rivlin (five-parameter) scheme, due to its strong correlation with in vitro mechanical testing results, is used (Kumar and Rao 2016), which has also been used by Carpenter et al. (2021)

14 $${W}_{s}={C}_{10}\left({\overline{I} }_{1}-3\right)+{C}_{01}\left({\overline{I} }_{2}-3\right)+{C}_{20}{\left({\overline{I} }_{1}-3\right)}^{2}+{C}_{11}\left({\overline{I} }_{1}-3\right)\left({\overline{I} }_{2}-3\right)+{C}_{02}{\left({\overline{I} }_{2}-3\right)}^{2}+\frac{1}{d}{\left( J-1\right)}^{2},$$

where ${C}_{10}$, ${C}_{01}$, ${C}_{20}$, ${C}_{11}$, and ${C}_{02}$ are the material constants, $d$ is the material incompressibility parameter, $J$ denotes the elastic deformation gradient determinant, and ${\overline{I} }_{1}$ and ${\overline{I} }_{2}$ represent the first and second Cauchy–Green deformation tensor invariant as (Kumar and Rao 2016)

15 $$\overline{I}_{2} = \left( {J^{{{\raise0.5ex\hbox{$\scriptstyle { - 4}$} \kern-0.1em/\kern-0.15em \lower0.25ex\hbox{$\scriptstyle 3$}}}} } \right)I_{2} ,$$

in which ${I}_{2}$ is the second invariant of strain tensor written as (Kumar and Rao 2016)

$${I}_{1}=\left({\lambda }_{1}^{2}+{\lambda }_{2}^{2}+{\lambda }_{3}^{2}\right),$$

16 $${I}_{2}=\left({\lambda }_{2}^{2}{\lambda }_{3}^{2}+{\lambda }_{1}^{2}{\lambda }_{2}^{2}+{\lambda }_{3}^{2}{\lambda }_{1}^{2}\right),$$

where $\lambda$ is the principal stretch. The material constants of the Mooney–Rivlin (5-parameter) hyperelastic model, used in this paper for the artery, are given in Table 2.

**Table 2:** Five-term Mooney–Rivlin model material constants (Holzapfel et al. 2005)

	C_10_ (MPa)	C_01_ (MPa)	C_20_ (MPa)	C_11_ (MPa)	C_02_ (MPa)	D (Pa^−1^)
First layer	− 0.19	2.03	11.30	− 0.19	20.10	1 × 10^−5^
Second layer	− 0.17	0.21	5.02	− 1.88	13.5	1 × 10^−5^

To consider the viscoelastic behaviour as an internal energy dissipation mechanism, a five-term Prony shear relaxation model is used. The Cauchy stress is written as (Haupt and Lion 2002; Karimi et al. 2017)

17 $$\sigma ={\int }_{0}^{t}2G\left(t-\tau \right)\frac{\text{d}e}{\text{dt}}d\tau +I{\int }_{0}^{t}K\left(t-\tau \right)\frac{\text{d}\Delta }{d\tau }d\tau ,$$

in which $\Delta$ is the volumetric strain, $e$ denotes the deviatoric strain, $I$ is an identity tensor, and $G\left(t\right)$ and $K\left(t\right)$ are the Prony series and the Bulk relaxation moduli, respectively, which are expressed as (Haupt and Lion 2002)

18 $$G\left(t\right)={G}_{0}\left[{\alpha }_{\infty }^{G}+\sum_{i=1}^{n}{\alpha }_{i}^{G}\text{exp}\left(-\frac{t}{{\tau }_{i}^{G}}\right)\right],$$

19 $$K\left(t\right)={K}_{0}\left[{\alpha }_{\infty }^{K}+\sum_{i=1}^{n}{\alpha }_{i}^{K}\text{exp}\left(-\frac{t}{{\tau }_{i}^{K}}\right)\right],$$

where ${G}_{0}$ and ${K}_{0}$ are the relaxation moduli at time equal to zero. $n$ is the number of terms, which is 5. ${\alpha }_{\infty }^{G}$ and ${\alpha }_{\infty }^{K}$ are the relative moduli. ${\tau }_{i}^{G}$ and ${\tau }_{i}^{K}$ are the relaxation times to develop the material coefficients. In this study, due to the lack of data regarding the bulk relaxation moduli, it is neglected (Carpenter et al. 2021). In Table 3, the relaxation time and relative moduli are provided for healthy coronary arterial walls as given in [51].

**Table 3:** Material parameters for the five-term Prony shear relaxation model (Karimi et al. 2017)

	i = 1	i = 2	i = 3	i = 4	i = 5
Relative moduli	6.28 × 10^−13^	0.53	7.15 × 10^−15^	0.47	3.67 × 10^−16^
Relaxation time (s)	10.90	60.85	585.55	627.35	886.51

It is also worth noting that alternative modelling strategies have also been proposed in the literature. For example, viscoporoelastic formulations have been employed to account for fluid–solid interactions within the arterial wall microstructure and to describe tissue adaptation and remodelling processes. Interested readers are referred to these studies for further details on viscoporoelastic approaches (Dell’Isola et al. 2019, 2009; Giorgio et al. 2016; Quiligotti et al. 2002).

### Geometry

**Fig. 1:**
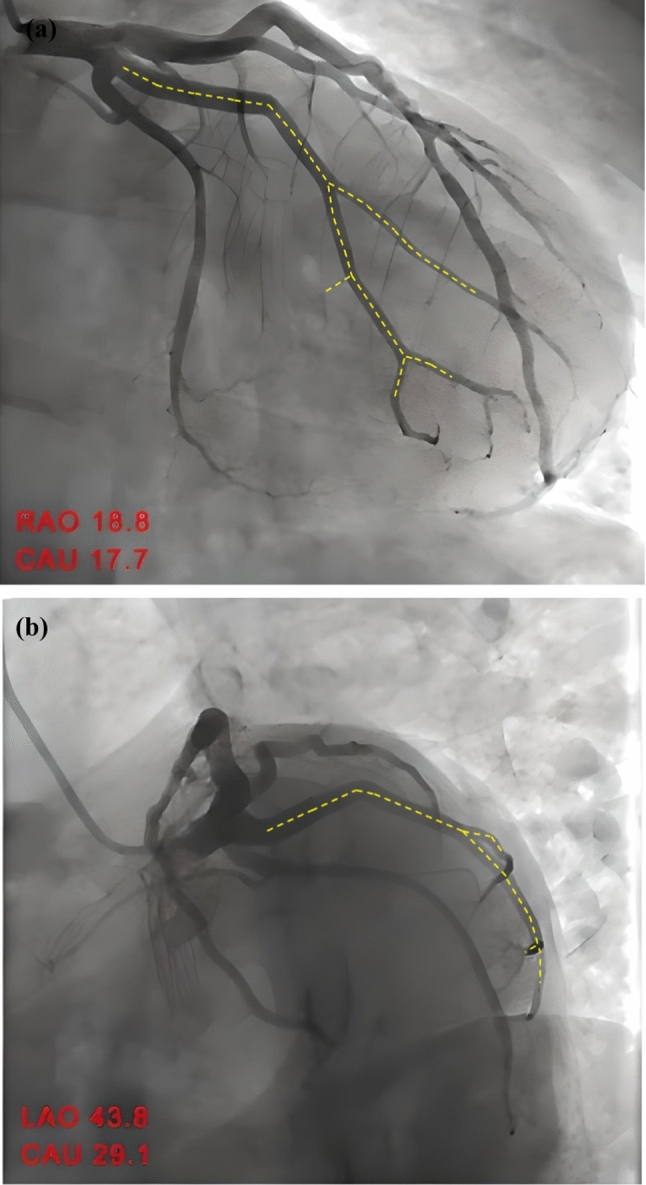
Coronary angiography images: (**a**) RAO 18.8 CAU 17.7 and (**b**) LAO 43.8 CAU 29.1 (Carpenter et al. 2021)

The geometry of the arterial system is reconstructed using clinical data obtained from angiography images provided by Carpenter et al. (Carpenter et al. 2021). This section details the development of both the lumen and artery models. Figure 1 shows the biplane angiography images for RAO 18.8, CAU 17.7 and LAO 43.8, CAU 29.1, highlighting the proximal and distal regions of the LCx artery.

**Fig. 2:**
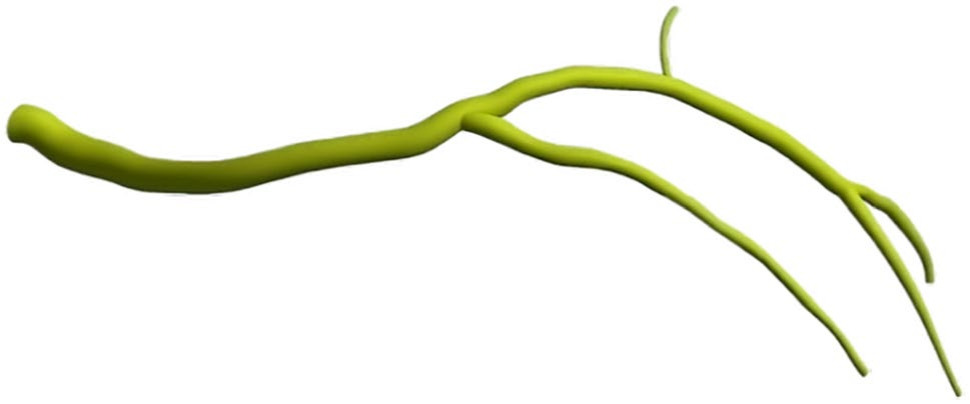
Finite volume model (Blood domain)

**Fig. 3:**
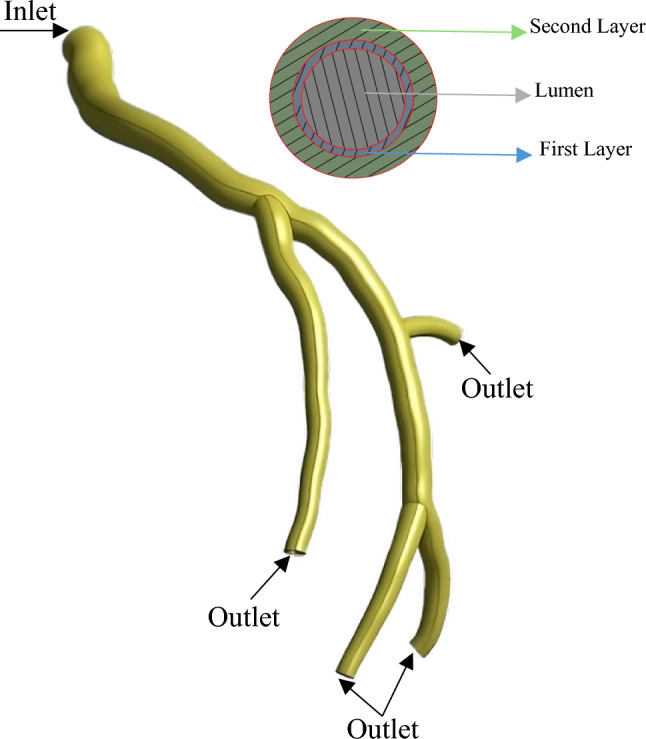
Finite element model (arterial wall structure)

The centreline of each plane is generated and projected normally from its respective plane. The three-dimensional centreline of the lumen is then constructed by intersecting the two projections. This procedure is repeated for each branch. After determining the 3D points of the lumen centreline, the corresponding edge points are extracted to calculate the cross section. Due to the lack of details regarding the shape of the artery in the angiography data, the cross section is assumed to be circular, calculated by averaging the edge points from two angiography images at two-millimetre intervals along the centreline. By importing the centreline and cross-section diameters into Autodesk Inventor (Autodesk Inventor 2025) and using the profiles-lofting technique, the final geometry is generated, as shown in Fig. 2. For the arterial wall thickness, values of 0.24 mm and 0.66 mm are chosen for the first and the second layers, respectively (Holzapfel et al. 2005). Figure 3 illustrates the final development of the artery reconstruction.

### Boundary conditions for the blood flow and the arterial wall

**Fig. 4:**
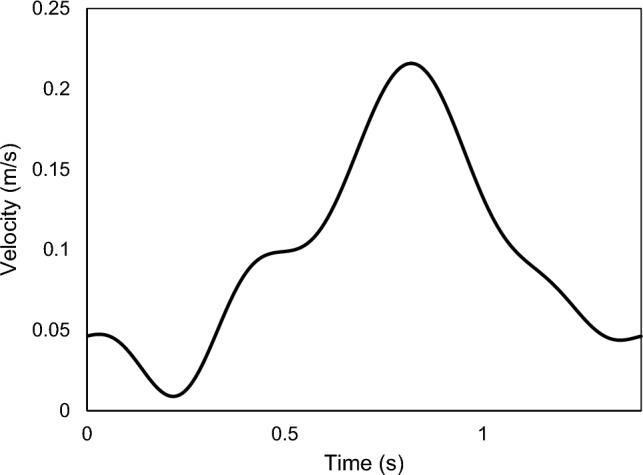
Inlet pulsatile coronary velocity profile for blood flow (Carpenter et al. 2021)

The inlet velocity is defined using the velocity profile provided by Carpenter et al. (Carpenter et al. 2021). Figure 4 illustrates the pulsatile blood flow inlet velocity profile for a heart rate of 43 bpm, corresponding to a cardiac cycle of 1.4 s. The pulsatile blood flow velocity is adjusted using an 11-term Fourier series to accurately represent the haemodynamics of blood flow, as described by Brigham (1988)

20 $$V\left(t\right)={\alpha }_{0}+\sum_{n=1}^{11}{\alpha }_{n}\text{cos}\left(\frac{2\pi nx}{T}\right)+{\beta }_{n}\text{sin}\left(\frac{2\pi nx}{T}\right),$$

where ${\alpha }_{0}$ is the mean value, $T$ is the period, $n$ is the number of terms, and $t$ represents time. The outlet pressure condition is set to a constant value of 8 kPa.

**Fig. 5:**
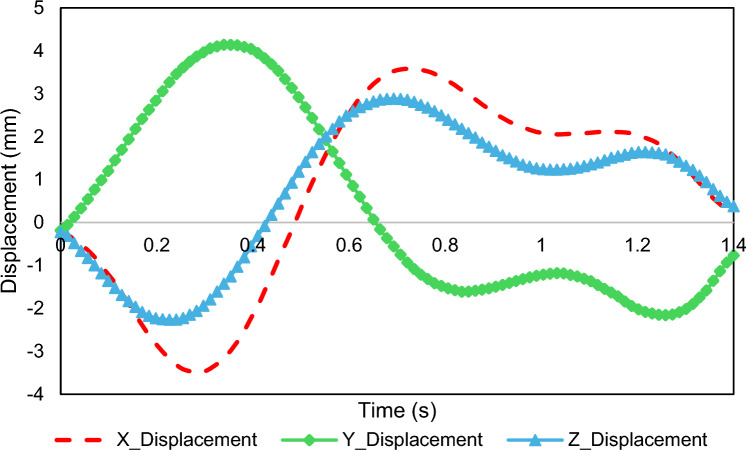
Boundary condition imposed artery's inlet and outlet surfaces (Carpenter et al. 2021)

The three-dimensional motion of the artery, induced by the cyclical motion of the heart chambers and respiratory dynamics, is incorporated into the model following the curve graph given in Fig. 5. An 11-term Fourier series is fitted to the displacement components and set to the inlet and outlet boundaries of the arterial wall.

To account for the muscular behaviour of the artery, constant radial pressure values of 3 kPa and 0.8 kPa are set for the outer surfaces of the first and second layers, respectively (Carpenter et al. 2021). No-slip boundary conditions are used for the plasma phase, and Johnson and Jackson boundary condition is applied to the RBC phase (Huang et al. 2009).

## Numerical simulations of two-phase FSI model

For the simulation of the nonlinear transient behaviour of the LCx coronary artery under a multiphase blood flow regime, ANSYS 2024R1 Workbench (ANSYS R12024 2024) with transient structural coupling to Fluent is selected. This set-up utilizes a two-way FSI approach, combining the finite element method (FEM) for the solid domain and the finite volume method (FVM) for the fluid domain. In this FSI approach, the Eulerian framework is employed to characterise the fluid flow, while the Lagrangian framework is used to describe the solid domain. The interaction between the solid and fluid domains at the interface is modelled using the arbitrary Lagrangian–Eulerian (ALE) technique. In the two-way FSI, the fluid flow is modified based on the structural deformations, along with the interaction between the FEM and FVM (Dhande and Pande 2017). To accurately model this interaction, dynamic mesh smoothing and remeshing are applied, in combination with the six-degrees-of-freedom solver in Fluent; this ensures proper control of the deformation and movement of the structures. For flow recirculation simulation over bifurcation regions, the k-ε standard viscous model is utilized, with standard wall functions utilized to establish the mesh in the viscous sublayer regions. In order to select an accurate and reliable element size, a comparative study is conducted in both the fluid and solid domains. In the fluid domain, four different mesh sizes (0.4, 0.2, and 0.17 mm) are compared, considering mesh quality parameters such as orthogonal quality and mesh skewness. In the solid domain, mesh sizes of 0.4, 0.3, 0.2, 0.18 and 0.17 mm are selected, and the von Mises stress and maximum principal stress values at the systolic time step are compared. Finally, a convergence is achieved with a mesh consisting of 1,019,404 elements with an average size of 0.17 mm for the fluid domain and 2,529,796 hybrid elements with an average size of 0.18 mm for the solid domain. To test the time independence of the solutions, 300 iterations are chosen. The residuals are set to 1 × 10^–4^ for continuity and granular temperature and 1 × 10^–6^ for the other variables. The coupled solver scheme is used to solve the momentum and continuity equations concurrently to preserve strong and accurate convergence for the complex flow conditions. After validating the time step, a time step of 0.007 s is selected for this study. Numerical simulations are conducted over 600 time steps, with a constant time step of 0.007 s, corresponding to a total of 4.2 s, i.e. three cardiac cycles. The results are derived from the final cycle to suppress the impact of flow instability at the beginning of the computation. A limitation of this study is that the initial arterial wall state was assumed to be stress-free, as patient-specific residual stress data were not available. However, by running three consecutive cardiac cycles and reporting results from the last cycle, the model incorporated the stresses generated during the first two cycles as an effective pre-stress state.

## Results based on the two-phase FSI model

In this section, results for the flow characteristics (i.e. RBCs volume fraction, velocity profiles, pressure distribution of blood mixture, and RBCs distribution), structural deformation, and the von Mises stress distribution are analysed. Additionally, haemodynamic parameters such as the time-averaged wall shear stress (TAWSS) and the oscillatory shear index (OSI) are evaluated.

### Blood flow characteristics and haemodynamic parameters

**Fig. 6:**
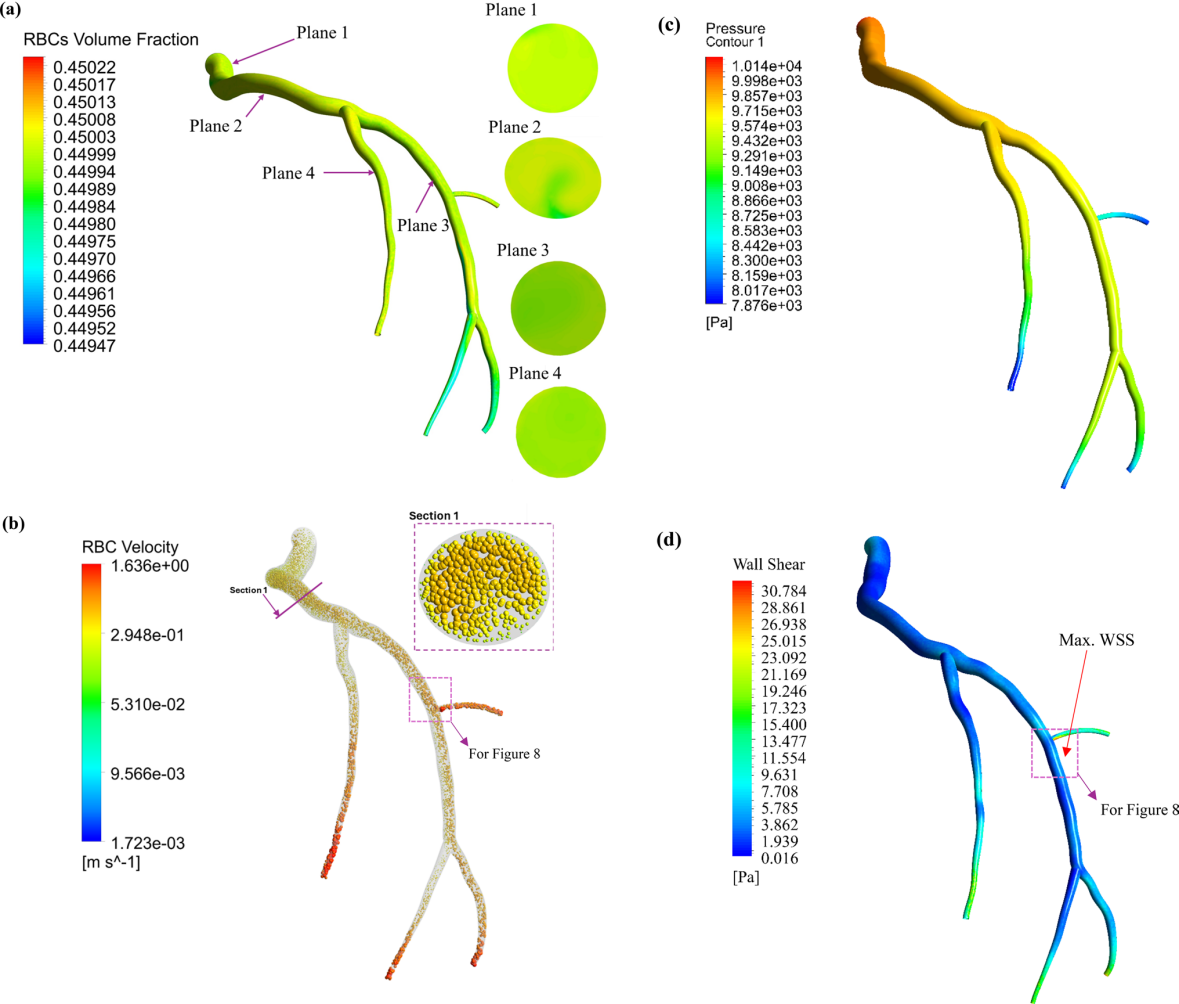
Blood flow characteristics: (**a**) calculated RBCs volume fraction distribution, (**b**) RBCs velocity vector, (**c**) pressure profile, and (**d**) wall shear stress (WSS) field within the LCx at the systole

Figure 6 presents the RBCs volume fraction, the velocity vectors of RBCs, the pressure profile, and the WSS field of LCx during the *systole* of the cardiac cycle. In this study, the two-phase blood model is considered with a 45% haematocrit concentration. The contour plots of the RBCs volume fraction throughout the LCx and at a cross-sectional plane along the main flow path of the LCx coronary artery are shown in panel (a) of Fig. 6. It is observed that the RBC volume fraction varies slightly along the LCx coronary artery, with the maximum variations at regions of curvature and sharp changes in cross-sectional area. The migration of RBCs is mainly influenced by the interaction between plasma and RBCs, as well as interactions between the RBC particles. The velocity vectors of RBCs obtained from the two-phase FSI model within the LCx coronary artery during the peak systolic phase are illustrated in panel (b) of Fig. 6. The velocity of the RBC particles reaches its highest magnitude near the centreline of the artery during both phases. The maximum RBC velocity, approximately 1.6 m/s, occurs in the narrowing regions of the artery. Near the arterial wall, the particle relative velocity is 0 m/s, while in the central region of the artery, it is approximately 0.29 m/s. In panel (c) of Fig. 6, the pressure profile of the LCx during the systolic is illustrated, where nearly 50% of the artery experiences a pressure range of 9500–10500 Pa, which aligns with the normal systolic blood pressure of a healthy human (i.e. less than 120 mmHg, or 16 kPa) (Sipahi et al. 2006). Wall shear stress (WSS) is associated with the tangential force exerted by the fluid flow on the wall that plays a key role in the occurrence and progression of intimal hyperplasia and atherosclerotic plaque formation. Figure 6 (d) shows the WSS field of LCx at the peak systole. The simulation shows that regions with smaller diameters, as well as bifurcated areas, experience elevated WSS likely due to the higher flow velocities in these zones.

**Fig. 7:**
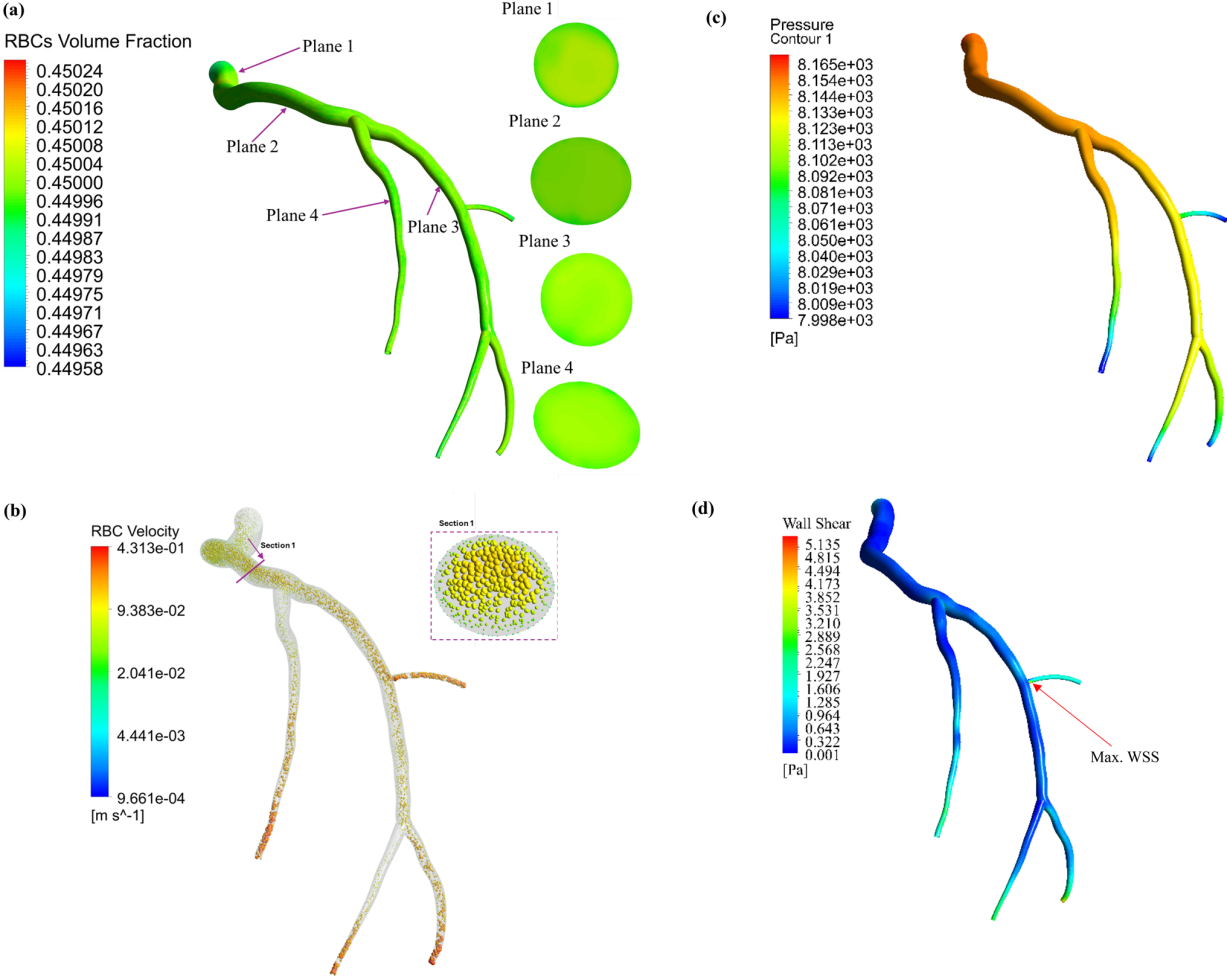
Blood flow characteristics: (**a**) calculated RBCs volume fraction, (**b**) RBCs velocity vector, (**c**) pressure profile, and (**d**) wall shear stress (WSS) field at the diastole

Figure 7 is the counterpart of Fig. 6 for the *diastole* of the cardiac cycle. As shown, during both the systolic and diastolic phases, the maximum WSS is observed in the same region near the bifurcation, as illustrated in panels (d), with values of 31.16 Pa and 4.37 Pa, respectively. As a result, these areas may be more susceptible to atherosclerosis. During the diastolic phase, the arterial pressure ranges from 8000 to 8165 Pa, which is consistent with the normal diastolic pressure in a healthy individual (Sipahi et al. 2006). As shown in Fig. 7a, the Fahraeus–Lindqvist effect, characterized by the migration of red blood cells away from artery walls, is more pronounced during the diastolic phase of the cardiac cycle.

**Fig. 8:**
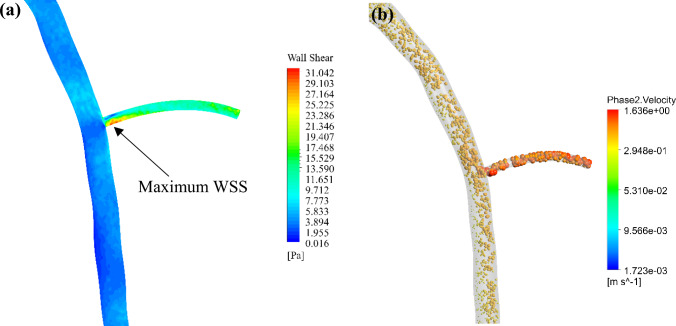
Comparison of (**a**) WSS profile and (**b**) RBC velocity vectors at the peak systole

Figure 8 is a zoomed snapshot of the regions shown in Fig. 6 (b, d). As seen, the maximum WSS area at the bifurcation correlates with the high velocity of the RBCs. This comparison provides valuable insight into the interaction between haemodynamic forces and the blood components.

Two main WSS-related haemodynamic indices, namely time-averaged WSS (TAWSS) and oscillatory shear index (OSI), which are mainly considered to assess the complex shear environment on the arterial wall, are analysed. TAWSS is calculated by averaging the WSS in a cardiac cycle as per Eq. (21), which is a better representation of WSS (Chiu et al. 2018). The OSI, as defined in Eq. (22), represents the oscillatory behaviour of WSS throughout the cardiac cycle; it is a dimensionless quantity and is more sensitive to detecting flow reversal than to variations in WSS magnitude over the cycle (Du et al. 2022).

21 $$\text{TAWSS}=\frac{{\int }_{0}^{T}\left|\overrightarrow{\tau }\right|dt}{{\int }_{0}^{T}dt},$$

22 $$\text{OSI}=0.5(1-\frac{\left|{\int }_{0}^{T}\overrightarrow{\tau }dt\right|}{{\int }_{0}^{T}\left|\overrightarrow{\tau }\right|dt}).$$

**Fig. 9:**
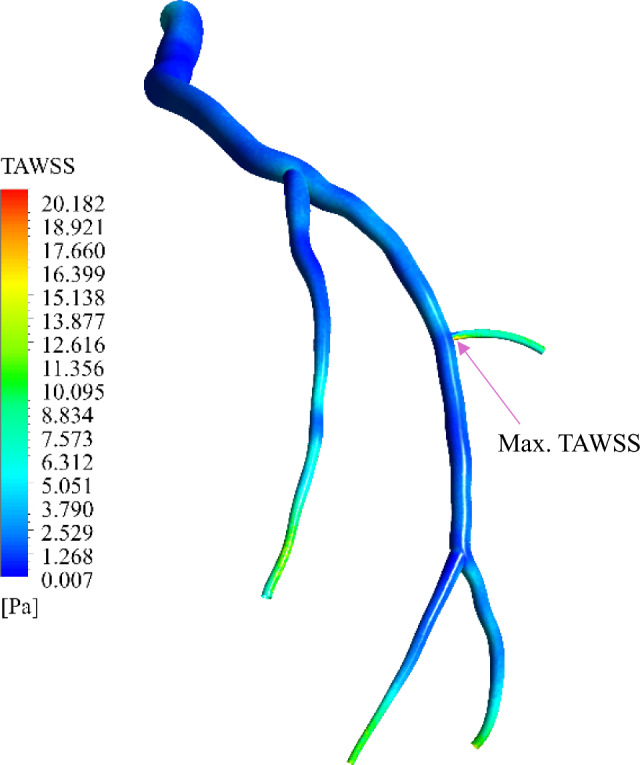
Variation of TAWSS along the LCx coronary artery

The TAWSS profile of the two-phase FSI model is presented in Fig. 9. From the contour, one can observe that there are very high TAWSS values at the bifurcation, where the blood flow is greatly decelerated, and a high wall velocity gradient is induced.

**Fig. 10:**
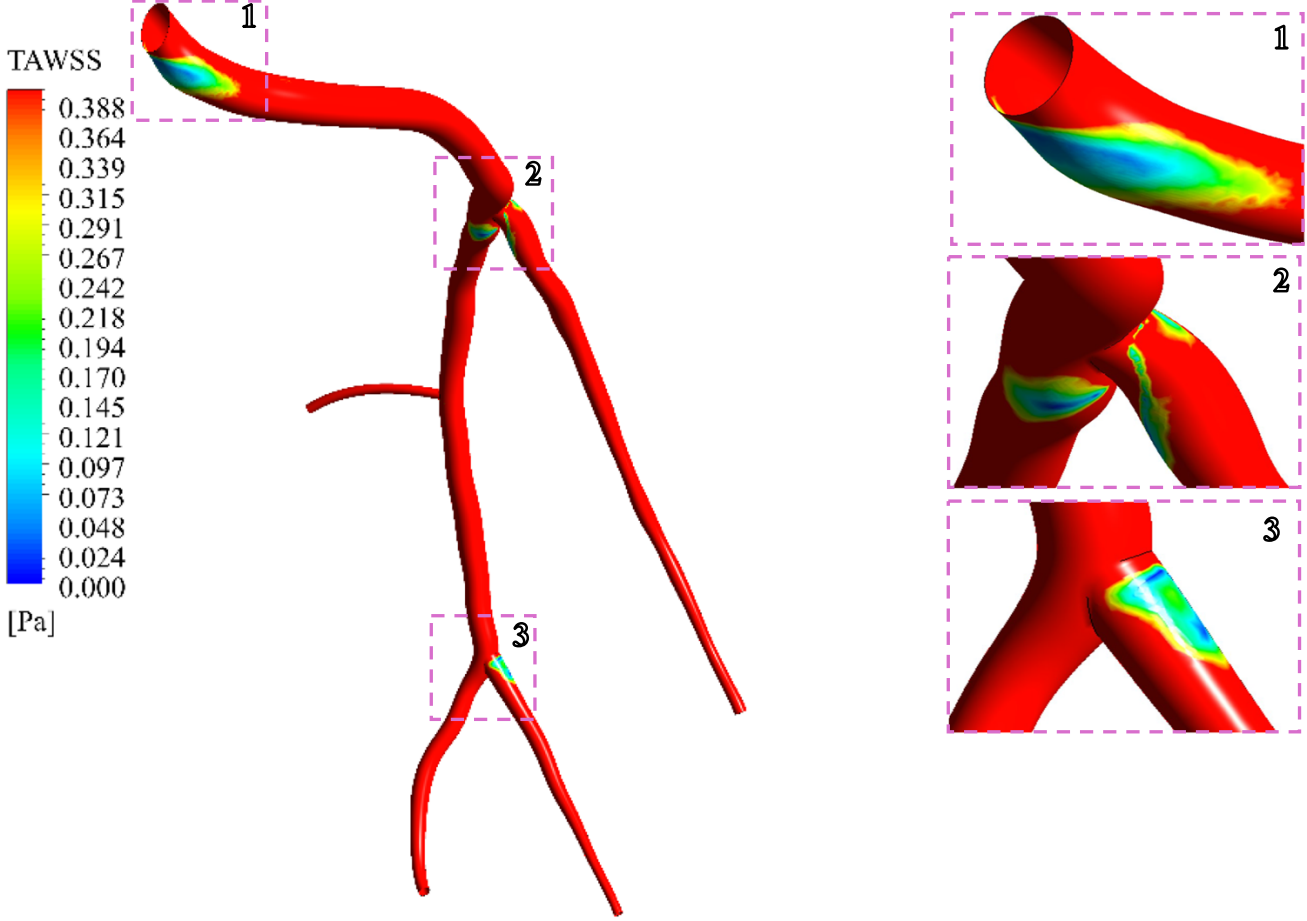
Time-averaged wall shear stress profiles—with TAWSS less than 0.4 Pa

According to Refs. (Buradi and Mahalingam 2020; Du et al. 2022), the locations with TAWSS values lower than 0.4 Pa are more susceptible to the formation of atherosclerosis. Figure 10 is a replot of Fig. 9, showing only the areas with the TAWSS values less than 0.4 Pa. As seen in the zoomed areas 1, 2, and 3, it can be concluded that the bifurcation area is more susceptible to developing stenosis, which can subsequently lead to atherosclerosis.

**Fig. 11:**
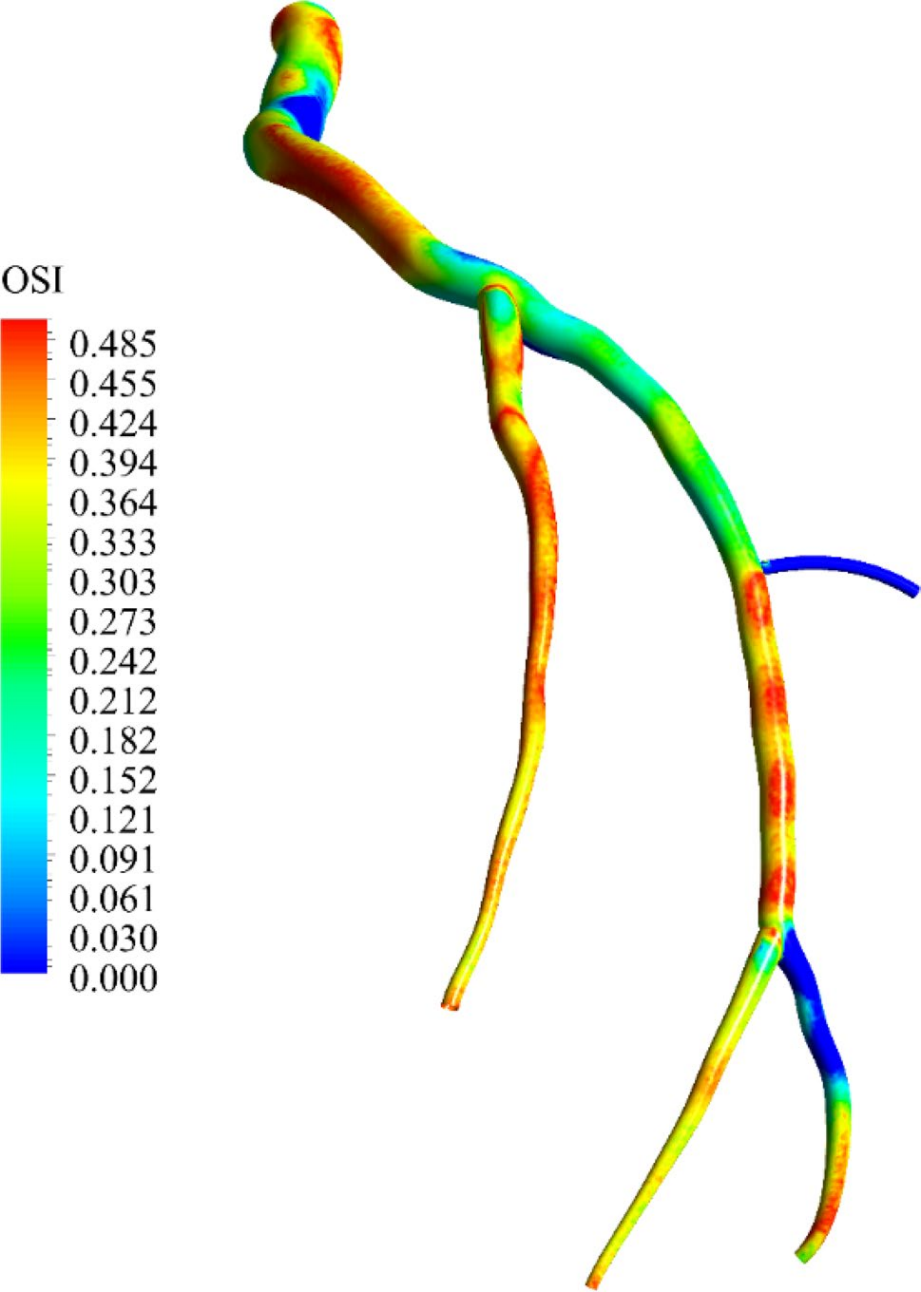
OSI contour, showing distribution of the OSI through the LCx artery

The dimensionless OSI is shown in Fig. 11. OSI is primarily used to identify areas of the artery where recirculation occurs (Schneiders et al. 2014). Based on numerous experimental studies (De Wilde et al. 2016; Ku et al. 1985), plaque formation tends to occur in regions where OSI is at its maximum, making OSI a valuable indicator for identifying such areas. As shown in Fig. 11, the OSI values are higher in the bifurcation areas, in regions with curvature, and in the areas with a relatively complex geometry; these regions are where blood flow slows down or reverses.

### Von mises stress profiling in arterial wall

**Fig. 12:**
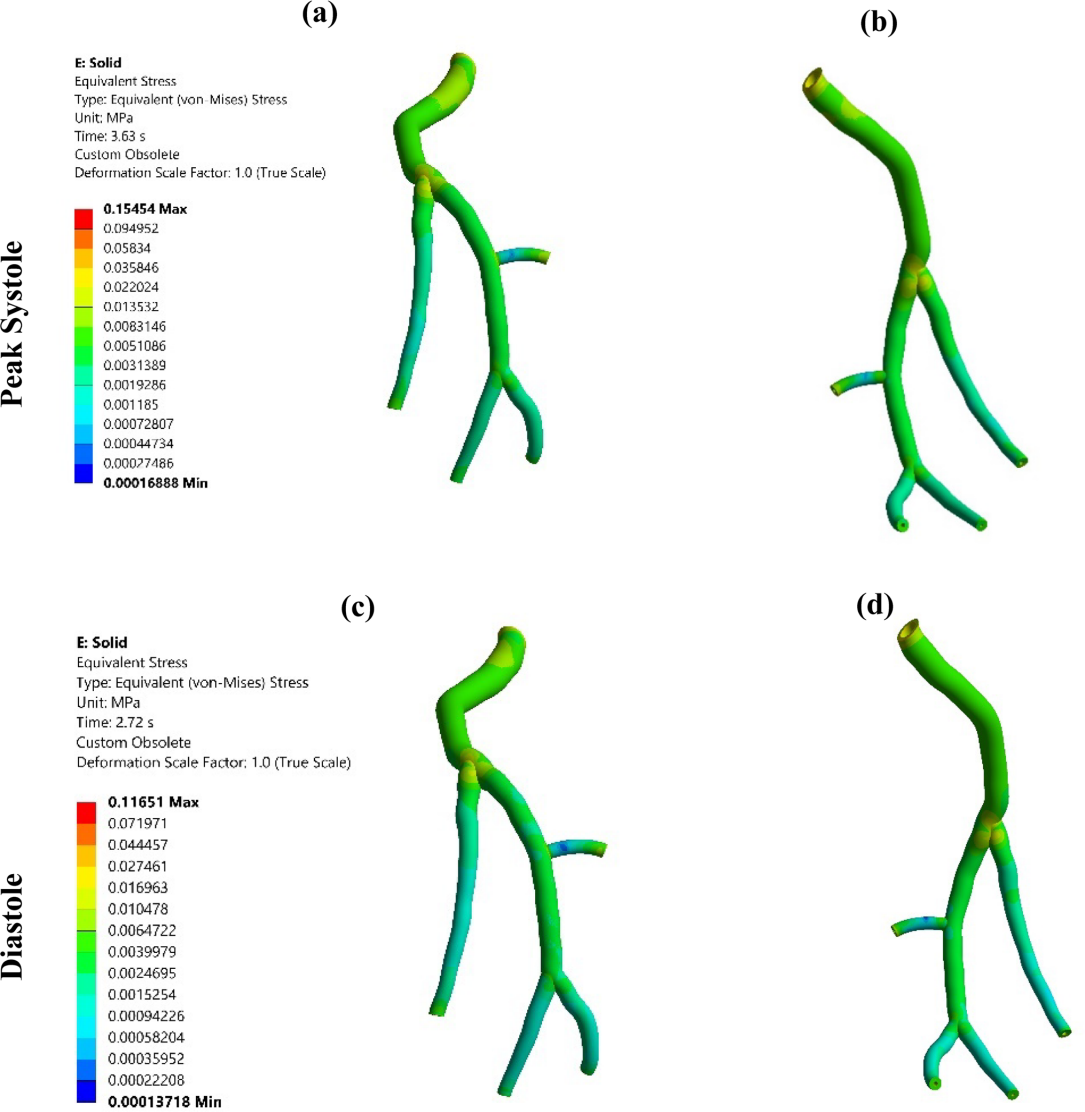
The von Mises stress distribution in the LCx coronary artery (**a**, **c**) Front view, (**b**, **d**) Back view

**Fig. 13:**
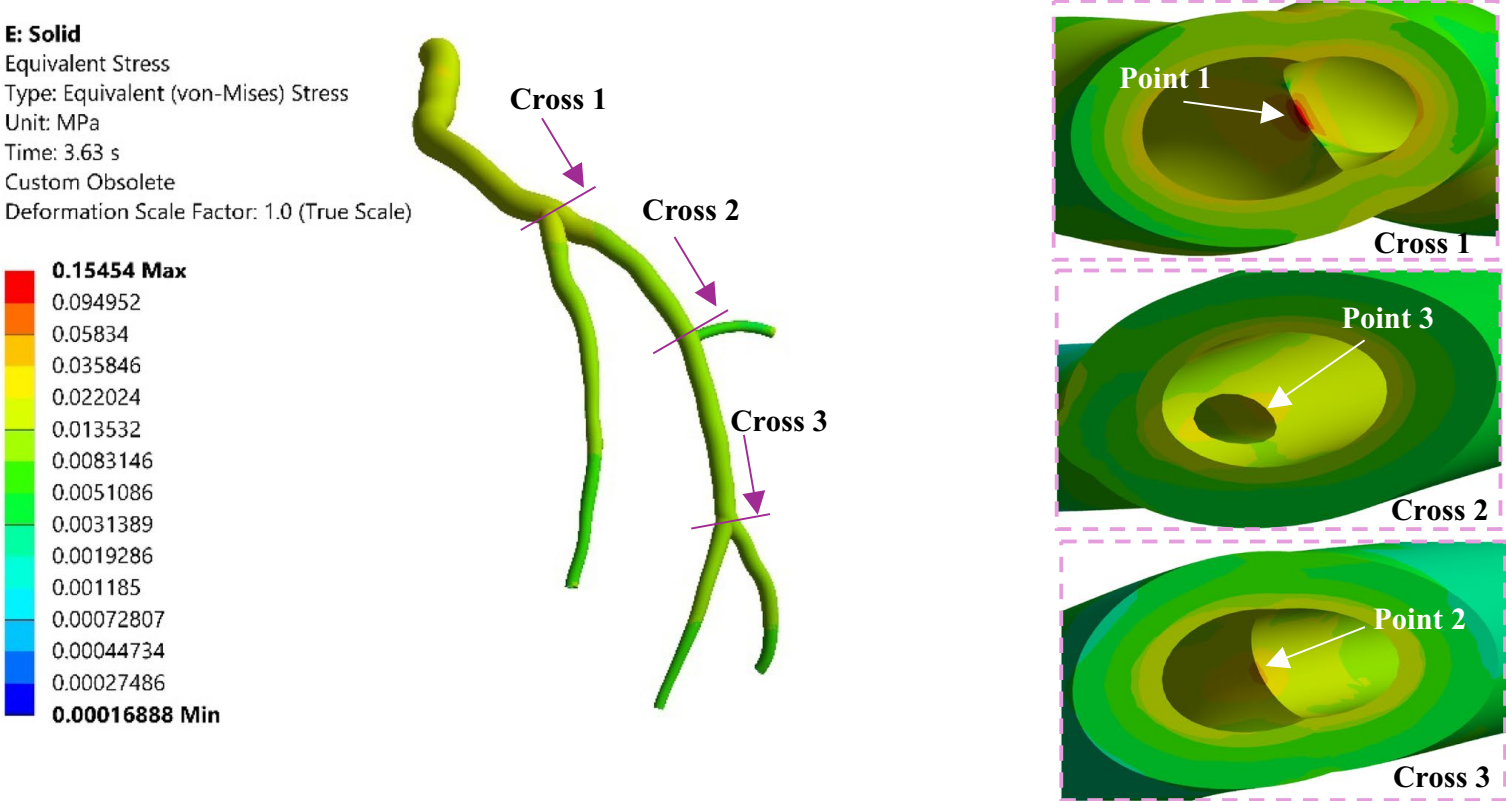
The VMS profile for both the intima and adventitia layers

The von Mises stress (VMS) is a mechanical parameter that represents the stress distribution within a structure, and it is mathematically expressed as (Mises 1928)

23 $$\text{VMS}=\sqrt{\frac{{\left({\sigma }_{1}-{\sigma }_{2}\right)}^{2}+{\left({\sigma }_{2}-{\sigma }_{3}\right)}^{2}+{\left({\sigma }_{3}-{\sigma }_{1}\right)}^{2}+6\left({\tau }_{12}^{2}+{\tau }_{23}^{2}+{\tau }_{31}^{2}\right)}{2}},$$

where ${\sigma }_{i}$ are principal normal stresses and ${\tau }_{ij}$ are principal shear stresses. The VMS distribution through the LCx artery is illustrated in Fig. 12 for the systole and the diastole; the cross-sectional views for the largest VMS magnitudes are shown in Fig. 13. The largest VMS magnitudes belong to the bifurcation areas (cross Sects. 1, 2, and 3). In addition, as illustrated by the cross sections in Fig. 13, the VMS in the second layer, i.e. media plus adventitia, is considerably lower than that in the first layer, i.e. intima plus transition zone between intima and media, which is consistent with previous studies. This observation supports the concept that the inner layer of the artery primarily bears the load under resting conditions, while the outer layer of the artery assumes the role of the main load bearer during high-stress scenarios (Carpenter et al. 2021).

**Fig. 14:**
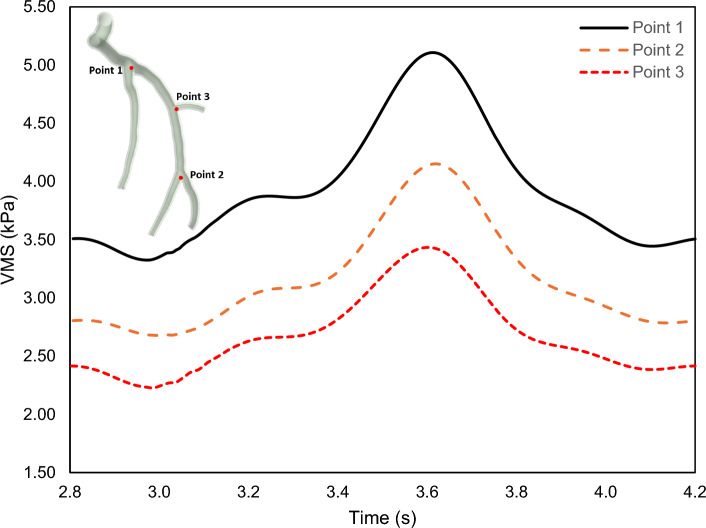
The von Mises stress variation at different locations of the LCx coronary artery at the last cardiac cycle

The time histories of the VMS at points shown in Fig. 13 are plotted in Fig. 14, highlighting that the largest belongs to Point 1 and the lowest is for Point 3; the time behaviour of all these points follows the same pattern.

## Effect of RBC representation (single-phase vs. two-phase) on haemodynamic measures

In this section, a direct comparison is made between the results of the two-phase FSI and the single-phase FSI models, i.e. with the presence of RBCs and single-phase approximation of blood flow. For the single-phase blood flow model, the power-law viscosity formulation is used as (Johnston et al. 2004)

24 $$\eta =k{\dot{\gamma }}^{n-1},$$

where *η* is the viscosity, $\dot{\gamma }$ denotes the strain rate, *k* is the consistency index, and *n* denotes the power-law index. The parameter values, used for the single-phase model i.e. without considering RBCs, are shown in Table 4.

**Table 4:** Parameter values used in the single-phase model for the power-law viscosity formulation (Johnston et al. 2004)

Density (*kg.m*^*−3*^)	1050
$k$	0.035
$n$	0.6

**Fig. 15:**
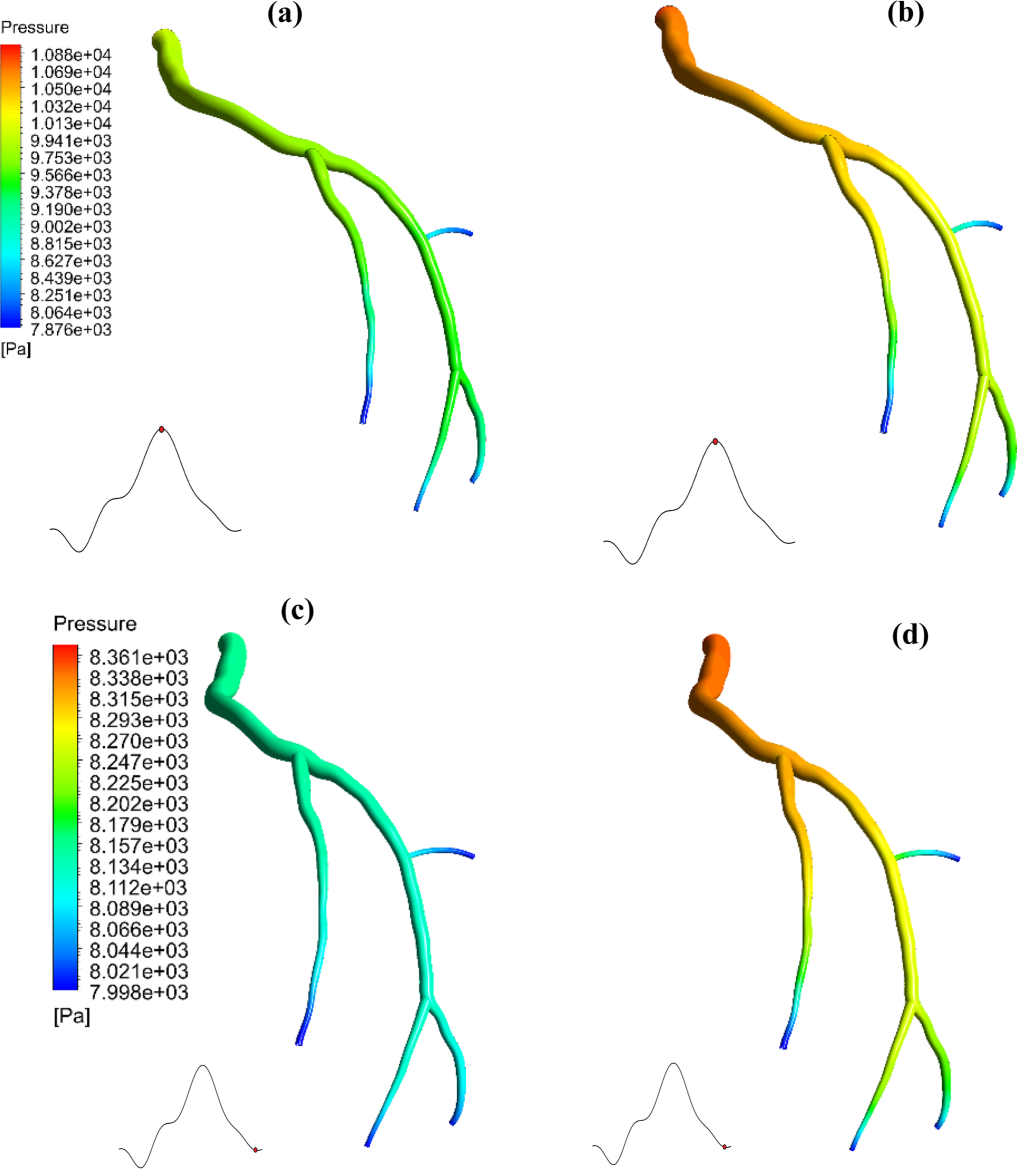
Wall pressure profiles in the LCx coronary artery during the systolic and diastolic phases of the cardiac cycle for (**a**, **c**) Considering RBCs, and (**b**, **d**) Single-phase (homogeneous) model

**Fig. 16:**
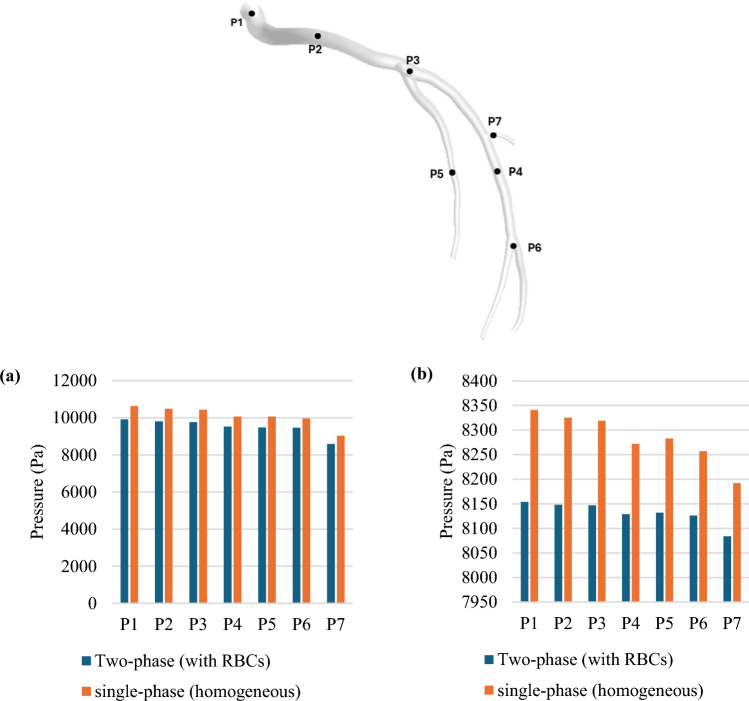
Pressure magnitudes collected from various points within the fluid domain (**a**) systole phase and (**b**) diastole phase

Figure 15 compares the wall pressure distributions of the two models: two-phase FSI and single-phase FSI for the LCx coronary artery during the systolic and diastolic phases of the cardiac cycle, i.e. panels (a,c) versus (b,d). The single-phase FSI model demonstrates higher-pressure magnitudes compared to the two-phase FSI model which considers the RBCs. To provide a more detailed comparison, pressure values are taken at various points along the arterial wall, as illustrated in Fig. 16; as seen, for both systole and diastole, the single-phase (homogeneous) model tends to overestimate the pressure values compared to the two-phase model.

**Fig. 17:**
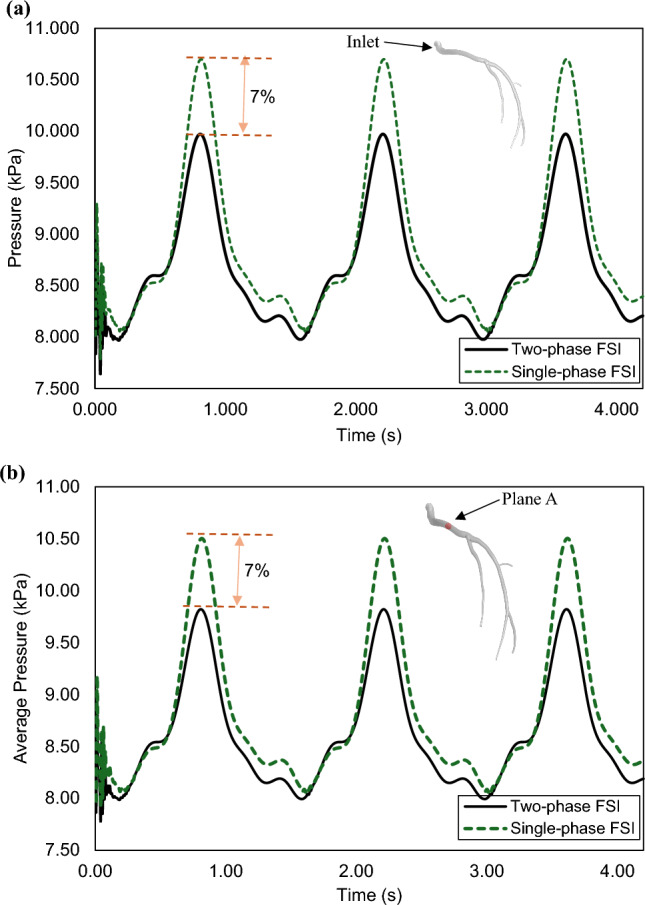
Pressure profile at (**a**) the inlet plane and (**b**) a plane in the main flow path through the cardiac cycle

The average pressures predicted based on the models, taking into account RBCs and ignoring them explicitly, are highlighted in Fig. 17, (a) at the inlet and (b) at Plane A (shown on the Fig. 17, (b)). As seen in both panels, the case with the RBCs shows lower average pressures.

**Fig. 18:**
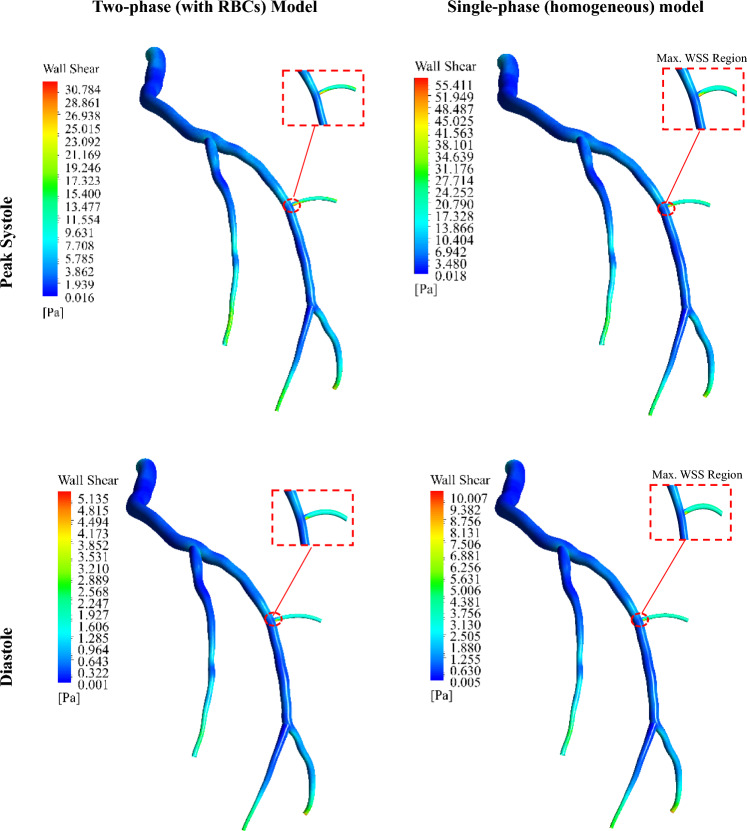
Wall shear stress (WSS) field in the LCx coronary artery during systolic and diastolic phases for two cases

Figure 18 addresses whether the location of the maximum wall shear stress (WSS) varies between the two models—with the presence of RBCs and single-phase approximation of blood flow. As shown, both models predict the same locations for the maximum WSS; however, the single-phase (homogeneous) model predicts a higher magnitude.

**Fig. 19:**
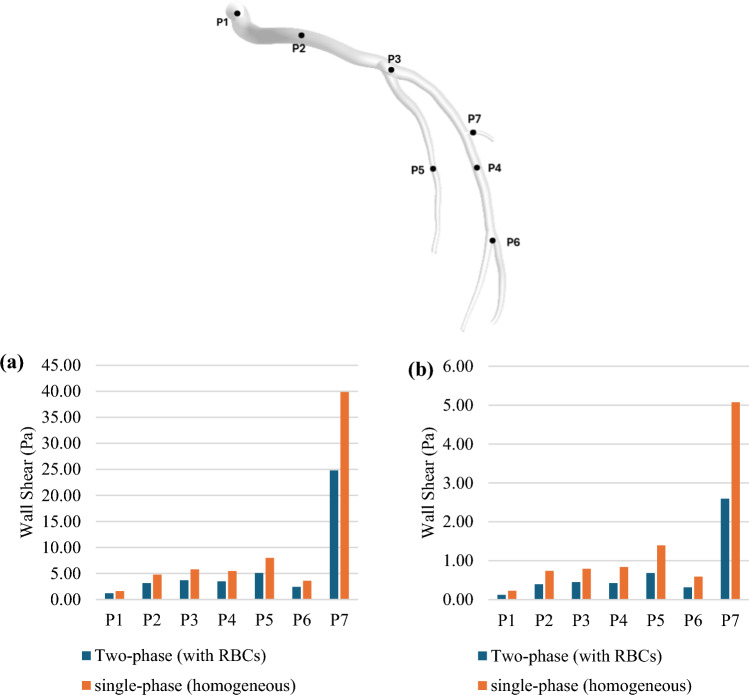
WSS magnitude taken from different points within the fluid domain (**a**) systole phase and (**b**)diastole phase

The variations in the WSS are examined in Fig. 19 for the two cases at two cardiac cycle phases, i.e. at the peak systole and the diastole. It is observed that the WSS values of the single-phase model are higher than those of the two-phase model at all points. At the location of maximum WSS (Point 7), the single-phase (homogeneous) model predicts WSS values approximately 60.7% and 95.4% higher than that of the model considering RBCs at the systole and the diastole, respectively.

**Fig. 20:**
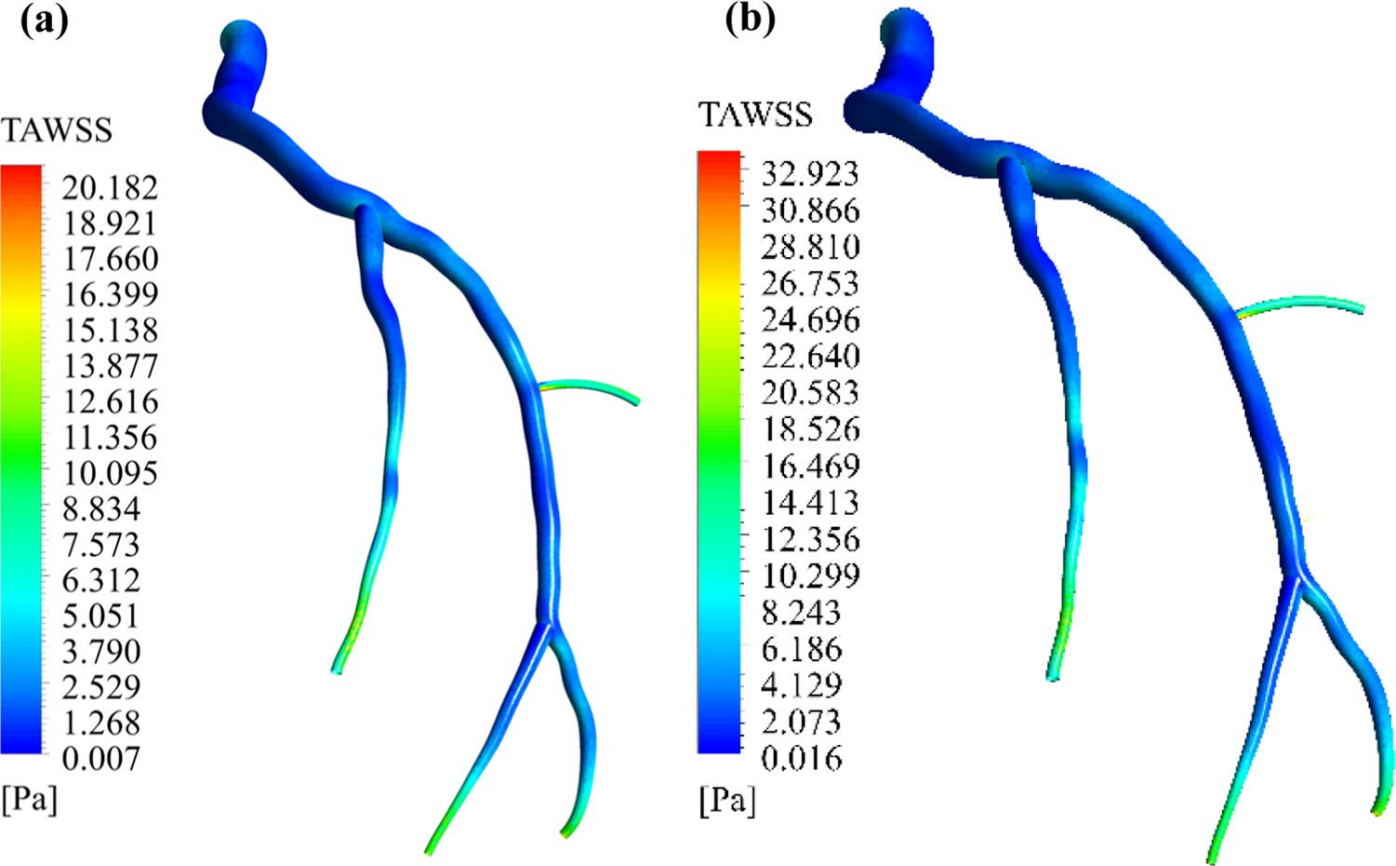
Time-averaged wall shear stress (TAWSS) field (**a**) two-phase FSI and (**b**) single-phase FSI

Comparing the TAWSS value in both cases, one can observe that the single-phase FSI model does forecast higher TAWSS values than the two-phase case (presence of RBCs), as shown in Fig. 20. It is also evident that the two cases predict the same vulnerable areas for potential atherosclerosis development; however, the two-phase FSI model predicts lower TAWSS magnitudes, leading to the identification of more susceptible zones than the single-phase FSI models.

**Fig. 21:**
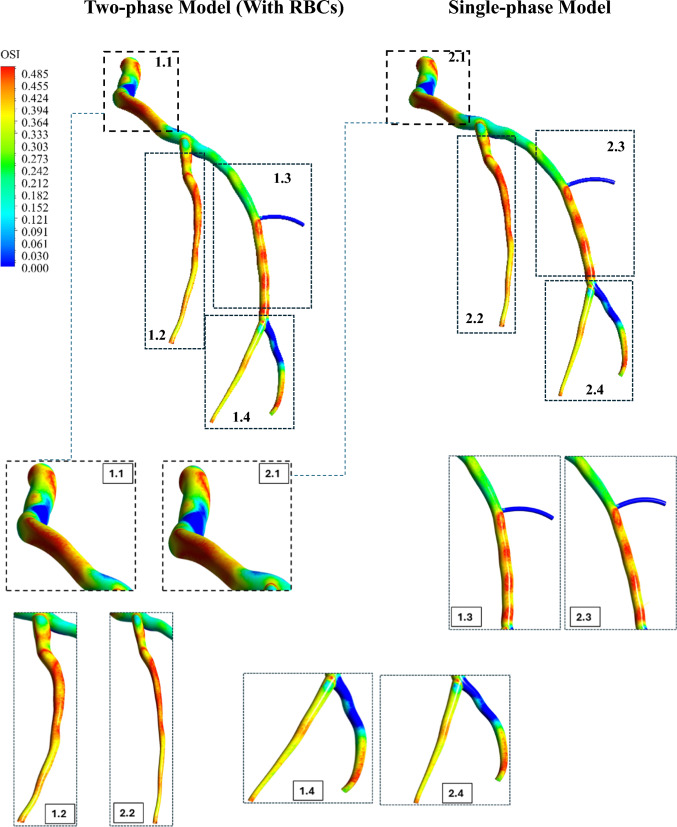
Visual comparisons of the OSI between the two-phase FSI and the single-phase FSI models

The dimensionless OSI is shown in Fig. 21 for the two models. As seen, both models predict quite similar regions of high OSI; however, overall, the model which considers RBCs explicitly as a separate phase predicts a slightly higher OSI.

In order to ensure a fair and comprehensive comparison between the single-phase and two-phase FSI simulations, we also performed an additional single-phase simulation using the Quemada viscosity model, which incorporates haematocrit as a parameter. While the power-law model was initially selected due to its extensive use in the literature for characterizing the shear-thinning behaviour of blood in large and medium-sized arteries, it does not directly account for the red blood cell (RBC) volume fraction. Since the two-phase FSI model in this study explicitly represents RBCs as a discrete phase (constituting 45% of the blood volume), employing a haematocrit-dependent viscosity law in the single-phase framework is crucial for ensuring a representative and meaningful comparison. The Quemada model describes the apparent viscosity of blood as a function of shear rate and haematocrit, thereby offering a more physiologically relevant description of blood rheology. The additional single-phase simulation with the Quemada model was carried out under the same boundary conditions, geometry, and numerical settings as the original power-law simulation, thereby isolating the effect of the viscosity model. The results from the Quemada simulations is presented in Appendix A, where the impact of incorporating haematocrit in the single-phase framework is quantified in terms of pressure distribution, wall shear stress indices, TAWSS and OSI.

## Concluding remarks

A complete biomechanical simulation for the determination of the biomechanical stress of the left circumflex (LCx) artery has been developed within the two-way FSI framework, considering blood flow as a two-phase flow composed of plasma and red blood cells (RBCs) with a haematocrit of 45%. To accurately capture the arterial wall response to the blood-induced pressure, viscohyperelastic properties have been employed. The key findings of this study, for the LCx artery considered, are outlined as:

- The two-phase two-way coupled FSI method used in this paper, using the kinetic theory of granular flow, confirms the Fahraeus–Lindqvist effect. The physical interpretation is that the RBCs tend to migrate away from the arterial wall, which is more pronounced during the diastolic phase.
- Pressure magnitudes are predicted to be lower when RBCs are considered explicitly, compared to the single-phase case; however, the velocity values remain unchanged between the two scenarios.
- A key haemodynamic value, the WSS, is predicted to be smaller for the two-phase model, i.e. in the presence of RBCs, compared to that of the single-phase model. This finding aligns consistently with that of previous studies (Huang et al. 2009).
- OSI, a significant variable providing informative data to health authorities, is larger when RBCs are considered explicitly, indicating larger oscillatory activity due to the interaction of blood phases.
- An additional significant haemodynamic value, the TAWSS, shows higher values when RBCs are not considered explicitly, i.e. the single-phase model. However, the locations of the maximum TAWSS do not change.
- Having blood as a two-phase medium (i.e. plasma and RBCs), while using a two-way coupled FSI framework, has been shown to be an important step in the advancement of numerical techniques for the prediction of cardiovascular events.

## Figures and Tables

**Fig. 22:**
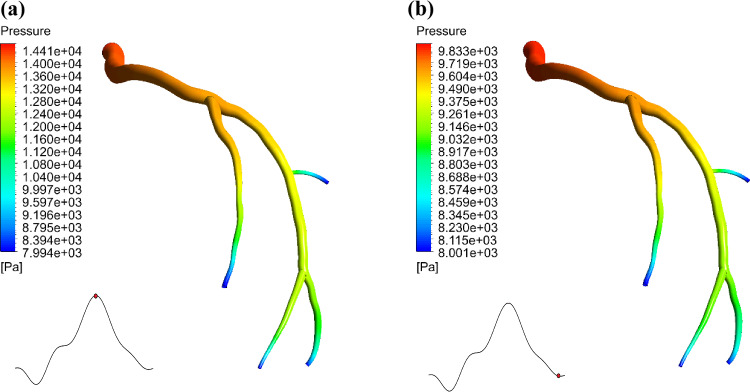
Wall pressure profiles in the LCx coronary artery during (**a**) the systolic and (**b**) diastolic phases of the cardiac cycle for single-phase case with Quemada viscosity model

**Fig. 23:**
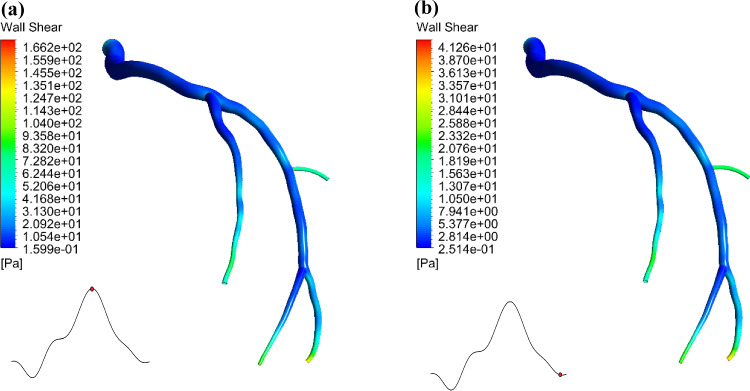
Wall shear stress (WSS) field in the LCx coronary artery during (**a**) systolic and (**b**) diastolic phases for single-phase case with Quemada viscosity model

**Fig. 24:**
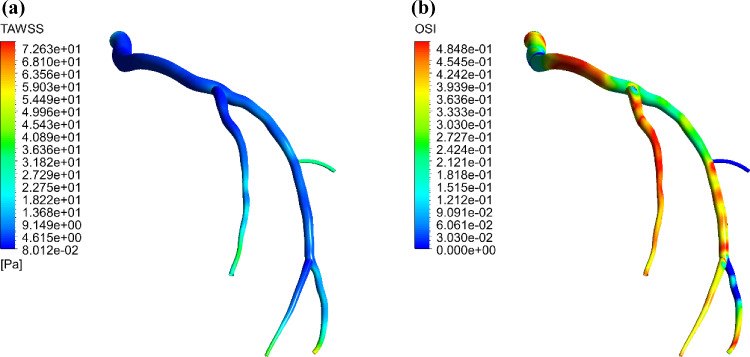
WSS-related haemodynamic indices: (**a**) time-averaged wall shear stress (TAWSS) field and (**b**) the OSI for the single-phase case with Quemada viscosity model

## Data Availability

This paper is a part of an ongoing research and hence the data cannot be shared at this stage.

## Appendix A

An additional single-phase FSI simulation is performed using the Quemada viscosity model, which explicitly incorporates the shear-thinning property with haematocrit variation on blood rheology. This model has been widely used to describe the non-Newtonian behaviour of blood in micro- and macro-circulation and offers a more physiologically representative alternative to the power-law model for single-phase simulations. The shear-thinning property of Quemada viscosity model is represented by the following constitutive model (Quemada 1978a, 1978b):

25 $$\mu \left(\dot{\gamma },\alpha \right)={\mu }_{P}{\left[1-\frac{\alpha }{2}\frac{{C}_{0}+{C}_{\infty }\sqrt{\dot{\gamma }/{\dot{\gamma }}_{c}}}{1+\sqrt{\dot{\gamma }/{\dot{\gamma }}_{c}}}\right]}^{-2},$$

where $\mu \left(\dot{\gamma },\alpha \right)$ is the variable blood viscosity, ${\mu }_{P}$ is the plasma viscosity, $\alpha$ is the RBC concentration, $\dot{\gamma }$ is the shear rate, ${\dot{\gamma }}_{c}$ is the critical shear rate, and ${C}_{0}$ and ${C}_{\infty }$ are viscosity coefficients. The parameter values, used for the Quemada viscosity model, are shown in Table 5.

**Table 5:** Parameter values used in the single-phase model for the Quemada viscosity model (Elhanafy et al. 2020)

Density (*kg.m*^*−3*^)	1050
${\mu }_{P}$(*Pa.s*)	1. 32 × 10^–3^
$\alpha$	0.45
${C}_{0}$	4.33
${C}_{\infty }$	2.07
${\dot{\gamma }}_{c}$(*s*^*−1*^)	1.88

Figure 22 shows the pressure contour plot of the LCx artery based on the Quemada viscosity blood flow model. This model predicts higher pressure values compared to the power-law model. For example, at peak systole the average inlet pressure is 14.3 kPa with the Quemada model, whereas it is 10.69 kPa with the power-law model, and 9.95 kPa in the two-phase model with RBCs explicitly considered—i.e. about 43.7% higher than the two-phase model.

Figure 23 illustrates the wall shear stress (WSS) field within the LCx during systole and diastole. This model predicts the locations of maximum and minimum WSS regions similarly to the power-law and two-phase models, but with higher values at the maximum regions. For example, at point 7 (as shown in Fig. 19), the WSS magnitude reaches 143.43 Pa at peak systole and 32.07 Pa at diastole. At point 3, the WSS magnitude is 9.65 Pa at peak systole and 1.23 Pa at diastole. 

Figure 24 shows the WSS-based haemodynamic metrics, TAWSS and OSI, obtained from the Quemada viscosity model. The spatial distribution of maximum and minimum regions is consistent with the power-law and two-phase models. However, the Quemada model predicts more pronounced high-OSI regions and higher TAWSS magnitudes compared to both the single-phase power-law and two-phase models.
